# Monitoring health interventions – who's afraid of LQAS?

**DOI:** 10.3402/gha.v6i0.21921

**Published:** 2013-11-08

**Authors:** Lorenzo Pezzoli, Sung Hye Kim

**Affiliations:** 1 Freelance Epidemiology Consultant, London, UK; 2 International Vaccine Institute, Seoul, the Republic of Korea

**Keywords:** lot quality assurance sampling, coverage, health interventions, immunization, meningitis, Niger

## Abstract

Lot quality assurance sampling (LQAS) is used to evaluate health services. Subunits of a population (lots) are accepted or rejected according to the number of failures in a random sample (*N*) of a given lot. If failures are greater than decision value (*d*), we reject the lot and recommend corrective actions in the lot (i.e. intervention area); if they are equal to or less than *d*, we accept it. We used LQAS to monitor coverage during the last 3 days of a meningitis vaccination campaign in Niger. We selected one health area (lot) per day reporting the lowest administrative coverage in the previous 2 days. In the sampling plan we considered: *N* to be small enough to allow us to evaluate one lot per day, deciding to sample 16 individuals from the selected villages of each health area, using probability proportionate to population size; thresholds and *d* to vary according to administrative coverage reported; *α* ≤5% (meaning that, if we would have conducted the survey 100 times, we would have accepted the lot up to five times when real coverage was at an unacceptable level) and *β* ≤20% (meaning that we would have rejected the lot up to 20 times, when real coverage was equal or above the satisfactory level). We classified all three lots as with the acceptable coverage. LQAS appeared to be a rapid, simple, and statistically sound method for in-process coverage assessment. We encourage colleagues in the field to consider using LQAS in complement with other monitoring techniques such as house-to-house monitoring.

When providing health services, such as vaccination (e.g. polio or meningitis vaccine), drugs (e.g. vitamin A or antihelmintics), or other interventions (e.g. mosquito nets), monitoring the extent of delivery is as important as the delivery itself, both during and after the process. In low- and middle-income countries, these field evaluations significantly absorb the already scarce human and financial resources but do not always produce useable results (1).

Lot quality assurance sampling (LQAS) is a quality control survey methodology, originated from the manufacturing industry, which has also been used to evaluate health services (2). Subunits of a population (lots) are accepted or rejected according to the number of failures in a random sample (*N*) of a given lot. If failures are more than *d*, the decision value, we reject the lot (i.e. the intervention area) and recommend corrective actions; if they are equal to or less than *d*, we accept it. If the objective is to simply classify lots, then LQAS can be very rapid, because sampling can be truncated as soon as the number of failures exceeds *d* or a sample of *N*–*d* without a failure is achieved, rather than collecting the full sample of *N*. In the statistics literature this is known as ‘curtailed’ sampling (3). The decision value, *d*, is determined by consideration of *N*, two coverage thresholds – the lower threshold (LT) or minimum acceptable threshold and the upper threshold (UT) or desired coverage threshold–, and acceptable probabilities of classification error (*α* and *β*). *α* (or consumer risk) is the risk of accepting a lot when coverage is unacceptable, meaning that the population is not provided with a service of acceptable quality (i.e. high coverage) but the area will be considered to have achieved the target coverage. *β* (or provider risk) is the risk of rejecting the lot when coverage is of acceptable quality, meaning that additional interventions will be recommended in an area where a sufficient proportion of the target population has been already provided with the service.

Generally, for coverage assessments, *α* is calculated from the LT and *β* from the UT. Therefore, LQAS performs well (errors are equal or below the expected) when classifying lots with coverage that is at either extremes, as shown by the operating characteristic (OC) curve, which plots the binomial cumulative distribution as an exact function of coverage (Fig. 1). When real coverage is neither high nor low, but moderate (i.e. between UT and LT), the classification will be less precise. The probability of a lot with a moderate coverage being classified as high coverage or low coverage is approximately proportional to the proximity of the coverage in that area to the classification thresholds (4). This methodological characteristic is also one of the main advantages of LQAS, because when the interval between the two thresholds (also known as ‘grey area’) is wide enough, the classification errors will be low even with a very a small *N* (5, 6). Logically, this advantage comes with the cost of increasing the area of uncertainty of our classification.

**Fig. 1:**
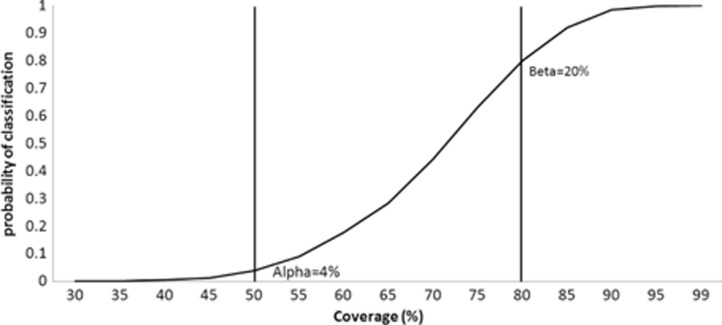
Operating characteristic curve for lot quality assurance sampling (LQAS) rule rejecting programmes with more than four defectives (*d=*4) in a sample of 16 (*N=*16), two vertical lines mark the lower threshold (LT=50%) and the upper threshold (UT=80%), Niger, December 2010.

Despite its advantages in terms of sample size, and rigorous action-oriented methodology, LQAS is used much less than other ‘traditional’ house-to-house monitoring techniques, which are mainly based on convenience sampling (7, 8). Is this because users are intimidated by the unfamiliar statistical concepts of LQAS?

‘Convenience’ approach definitely sounds more appealing and simple. However, this term is intended as the non-probabilistic (thus non-generalizable) selection of units at-risk of low coverage, rather than as an easy and effortless way of sampling.

In order to explore alternatives for house-to-house monitoring, we used LQAS to monitor coverage before the end of a 10-day meningitis vaccination campaign conducted in Niger in December 2010 (9). During the last 3 days of the campaign, we selected an area covered by one primary health centre (lot) per day reporting the lowest administrative coverage in the suburb of the capital city, Niamey (i.e. the number of vaccine doses administered divided by the number of target population in the area) in the previous 2 days. The sample size for each lot was calculated using the freeware software SampleLQ v1.10 (http://www.brixtonhealth.com/samplelq.html), based on cumulative binomial probabilities (10). For each LQAS plan we considered: *N* to be small enough to allow us to evaluate one lot per day, deciding to sample 16 individuals; thresholds and *d* to vary according to administrative coverage reported; *α* to be maximum 5% and *β* maximum 20%. We had one team of two surveyors (one female and one male, both familiar with the area surveyed and fluent in the local languages) with a driver and a vehicle. Each day they visited one health area and completed one lot. They selected the 16 random sampling points per lot by probability proportionate to population size (PPS) from the list of villages with population data available from the corresponding primary health centre. Once in the selected village, the surveyors chose the first household randomly using a geographic sampling procedure. This entailed drafting a map of the locality, dividing it into smaller sectors according to existing divisions (streets, rivers, etc.), and selecting one sector according to simple random sampling (SRS). Once a sector with approximately 20 houses or less was reached, one household was selected randomly. Only one individual eligible for the vaccine (i.e. aged between 1 and 29 years) was selected randomly in the house for the survey. In case we had to select more than one person per village (this could have happened in large villages since PPS was used), we selected subsequent households by repeating the abovementioned procedure. Each day's survey was truncated as soon as a sample of *N*–*d* without a failure was achieved, before the full sample had been collected.

Results of the three LQAS surveys are detailed in Table 1. We classified all three areas as with acceptable coverage. In case of rejection, we would have recommended to exercise extra efforts in vaccinating the population in the problem areas identified, before the end of the campaign, while logistics were still in place and control measures could have been implemented more easily and rapidly.

**Table 1:** LQAS sampling plans used day by day at health centre level during the meningitis A vaccination campaign, Niger, December 2010

Lot	Day of campaign	% Administrative coverage (at days of campaign)	*N*	*d*	LT%	UT%	*α* (%)	*β* (%)	*V*	*U*	Classification
A	8	69.44–77.71 (6–7)	16	4	50	80	4	20	12	0	Accepted
B	9	68.64–73.11 (7–8)	16	3	55	85	3	21	13	0	Accepted
C	10	75.24–77.72 (8–9)	16	2	60	90	2	21	14	0	Accepted

*N*, lot sample size; *d*, decision value; LT, lower threshold; UT, upper threshold; *V*, number of vaccinated individuals found; *U*, number of unvaccinated individuals found.

In our LQAS plans, we prioritized having lower *α* (i.e. the risk for the population) than *β* (i.e. the risk for the service providers). *α* of 5% or less meant that, if we would have conducted the survey 100 times, we may have classified a lot with acceptable coverage up to five times when real coverage was below an unacceptable level (LT). Conversely, *β* of 20% meant that we may have classified the lot as unacceptable coverage up to 20 times, when real coverage was above the satisfactory level (UT). These levels of error were achieved by having ‘grey areas’ of at least 30% and the sample size of 16. Such a level of uncertainty may not be appropriate for accurate post-campaign coverage assessment, but served our purpose well during the campaign, since we needed to assess coverage rapidly.

One possible limitation was that for simplicity of conduction the villages were chosen based on PPS instead of SRS of all residents in the lot. Although PPS is common practice in the field because it provides self-weighted samples, it could have introduced a certain degree of correlation affecting the LQAS errors, the effect of which is unknown.

LQAS appeared to be a rapid, simple to use, and statistically sound method for in-process monitoring of coverage. Bearing in mind the mantra ‘for better coverage, measure coverage better’ (11), we encourage colleagues in the field to consider using LQAS as an alternative to or in complement with traditional house-to-house monitoring tools.
